# HIV, smoking, and the brain: a convergence of neurotoxicities

**DOI:** 10.1186/s12981-025-00714-y

**Published:** 2025-02-06

**Authors:** Benjamin L. Orlinick, Shelli F. Farhadian

**Affiliations:** Yale School of Medicine, Section of Infectious Diseases, New Haven, CT USA

**Keywords:** HIV, Tobacco smoking, Neurocognitive impairment, nAChR, Neuroinflammation, Nicotine

## Abstract

**Purpose of review:**

The purpose of this review is to characterize the combined effects of tobacco smoking and human immunodeficiency virus (HIV) infection in people with HIV (PWH) and identify possible therapeutic targets through shared mechanisms for neurotoxicity.

**Recent findings:**

HIV and tobacco smoke can exert neurotoxicity through shared mechanisms such as brain volume changes, microglial dysregulation, and dysregulation of the cholinergic anti-inflammatory pathway (CAP) through the alpha7-nicotinic acetylcholine receptor (nAChR). Evidence also suggests the potential for synergistic effects of HIV and tobacco smoking on neurotoxicity.

**Summary:**

People with HIV (PWH) are disproportionately affected by both neurocognitive impairment (NCI) and tobacco smoking compared to the general population. Both HIV and tobacco smoking are known to have neurotoxic effects and have the potential for clinically significant impacts on brain health and cognitive functioning. Less, however, is known about how PWH may be uniquely affected by the interactive neurotoxic effects of both HIV and tobacco smoking. Evidence suggests that smoking and HIV can have synergistic effects on neurotoxicity and NCI in PWH. Some mechanisms for neurotoxicity include increased oxidative stress from microglial activation and dysfunction in the alpha7- nAChR cholinergic anti-inflammatory pathway leading to increased neuroinflammation and neuronal apoptosis. Mechanisms may vary by cell type and brain region, however, and there is a need for more human-facing, longitudinal studies on smoking in PWH.

## Introduction

Neurotoxic effects of tobacco smoking in people with HIV (PWH) is of increasing concern, given extremely high rates of tobacco smoking among PWH. The 2023 UNAIDS report estimates that, among 39.0 million PWH globally, 29.8 million are on ART, and there are estimated to be more than 4 million PWH who actively smoke [1, 2]. PWH are also more likely to smoke than those without HIV [2–7]. Estimates of smoking prevalence in PWH vary globally, with national estimates in the US and Italy as high as 50% compared to approximately 25% in the general population [3, 5]. In low- and middle-income countries, PWH are also more likely to smoke than people without HIV [6, 7]. Despite demonstrated willingness among many PWH to stop smoking, cessation can pose additional challenges in PWH [4]. Some possible explanations for this include faster nicotine metabolism in PWH, and high rates of self-medication for psychiatric illness such as anxiety and depression coupled with possible misconceptions about positive effects of smoking on T cell count and immune function in PWH [8–10].

In addition to higher rates of smoking, PWH also face higher rates of neurocognitive impairment (NCI) compared to the general population, with impairments affecting memory, attention, visuospatial skills, and other cognitive domains [11–15]. While there is much debate about the diagnostic criteria for and exact prevalence of NCI among PWH, HIV’s effects on the brain continue to be an important issue in the post-ART era, with an estimated 7 million PWH affected by some degree of symptomatic cognitive impairment [16–20].

In people without HIV, smoking associates with poorer cognitive function and there is evidence to suggest that HIV and tobacco smoking can have combined negative effects on brain structure leading to atrophy [21–26]. This is consistent with many other studies suggesting an additive or synergistic effect of smoking and HIV on NCI [25–31]. However, the mechanisms behind these combined effects are not well characterized. These possible mechanisms are complicated by the neuroprotective effect of acute nicotine exposure and how this effect may change with chronic exposure [32–36].

The purpose of this review is to explore how tobacco smoking in PWH may interact with HIV infection to affect the central nervous system (CNS), and to highlight possible shared mechanisms in HIV and smoking neurotoxicity that may serve as future therapeutic targets to improve brain health in PWH.

### Individual effects of HIV and tobacco use on neurocognitive impairment

#### HIV associations with neurocognitive impairment

NCI continues to disproportionately affect PWH despite effective ART regimens. However, due to high rates of comorbid substance use, coinfections, and heterogeneity of symptoms and symptom severity, classification of this HIV associated NCI as a unified disorder can be problematic [17, 18]. HIV associated NCI tends to affect several cognitive domains including memory, attention, visuospatial skills, speed of information processing, verbal fluency and motor skills [11–15]. Prevalence estimates for HIV associated NCI vary widely, and show significant heterogeneity both regionally and in subgroups of PWH affected [12–15, 20, 37]. It has been estimated that 72% of HIV associated NCI is in Sub-Saharan Africa [20]. 

#### Tobacco smoking associations with neurocognitive impairment

There is ample evidence to support that tobacco smoking associates with NCI and increased age-related cognitive decline in the general population. Compared to people who do not smoke, people aged 18–29 who smoke show significant impairment on tests of sustained attention, spatial working memory, executive planning, and were less likely to adjust behavior as a function of risk [24]. Longitudinal studies not only corroborate these findings, but also show increased rates of cognitive decline across age groups in people who smoke. Data from the Whitehall II cohort of British civil servants shows men, but not women, who currently smoke experience faster decline than men who do not smoke in measures of global cognition and executive function [23]. The reasons for this sex difference are not known but may be a function of the small number of women enrolled in this study. Similarly, using a prospective birth cohort, smoking was associated with more rapid decline in memory from age 43 to 53, despite similar baseline memory scores between people who smoke and those who do not at 43 [22]. Focusing an elderly cohort, a study examining people who smoke over 65 using Mini-mental state examination (MMSE) scores demonstrates higher rates of decline among those who smoke, though this is predominantly used as a dementia screening tool [21].

Fortunately, smoking cessation can ameliorate some of these cognitive deficits, but duration of smoking is an important factor to consider for extent of impairment [22, 23, 38]. Pack years, commonly defined as cigarettes per day divided by 20 times the number of years smoked, is associated with greater cognitive impairment [21, 38]. Greater pack-years also associate with more rapid brain aging among people who smoke and increased atrophy in the hippocampus [26, 38].

### Combined effects of smoking and HIV on neurocognitive impairment

Epidemiological studies assessing for a link between smoking and cognitive impairment in PWH has largely shown a negative association between smoking status and cognitive performance. In a moderately sized population of PWH (*n* = 125), most of whom were on ART (82%), Bryant et al. found current smoking in PWH is associated with worse outcomes in learning, memory, and global cognitive functioning [28]. When comparing people who smoke with and without HIV, people who smoked with HIV performed worse on assessments of working memory and processing speed, and exhibited higher intra-individual variability suggesting additive effects of HIV and smoking on cognition [31]. Evidence also suggests that HIV infection and chronic smoking may lead to additive deleterious effects on impulsivity and psychopathological symptoms in addition to cognitive dysfunction [30].

In addition to cognitive deficits, it is important to consider exacerbated psychiatric symptoms in PWH who smoke. These are crucial because psychiatric symptoms such as increased depression ratings correlate with lower neurocognitive performance in PWH who smoke [31]. PWH who smoke also score the highest on Center for epidemiologic studies-depression (CES-D) scale compared to people who smoke without HIV, and PWH who do not smoke, and seronegative controls even after controlling for the increased rate of antidepressant use and anxiolytics in PWH [3, 39].

While much of the available evidence suggests PWH experienced worse cognitive effects from smoking compared to people without HIV, some studies have found the opposite: that PWH who smoke do not have increased rates of cognitive impairment and are not at higher risk of cognitive decline [40–42]. The conflicting clinical evidence is summarized in Table 1 for reference. Wojna et al. conducted an observational cross-sectional study in a cohort of women with HIV (WWH). While WWH were more likely to report a history of smoking and have a higher plasma viral load and CD4 count, no correlations were observed between CSF viral load and smoking history or current smoking nor between cognitive impairment and current or history of smoking [40]. Interestingly, when the analysis was restricted to WWH, those who smoked tended to perform better on tests of psychomotor speed [40]. However, this study is limited by its cross-sectional, observational design and a small sample size with only 36 WWH. 24 of these WWH have some smoking history with 15 identifying as people who smoke currently. Furthermore, the authors do not examine neurocognitive differences among WWH based on smoking status despite finding evidence of perturbations in viral suppression and CD4 + T cell count due to smoking. Similarly, using a more limited measure of cognitive function, Tsima et al. also found no evidence for a link between smoking and clinically significant cognitive impairment in PWH [42]. While Tsima et al. performed analyses on a large cohort, the neuropsychological testing battery was limited, consisting only of the Mental Attention Test (MAT). This battery has participants count sequentially from 1 to 20, recite the alphabet, and repeat alternating between numbers and letters for 30 s, which may not adequately detect mild or asymptomatic NCI. Additionally, due to the retrospective nature of this study, investigators were unable to examine smoking duration and quantitative smoking frequency, and sensitivity analyses revealed a significant association between smoking and cognitive function when considering people who reported smoking every day versus not at all [42]. Finally, In a longitudinal analysis of the all-male Multicenter AIDS Cohort Study, no significant differences in the rates of neurological decline was observed among never smokers, former smokers, and current smokers when treated as categorical variables in PWH; these findings were similar to those observed in the HIV uninfected control group [41]. However, this study used a limited neuropsychological testing battery focusing only on domains of mental flexibility on processing speed, and did find a small but significant association between decline in these cognitive domains and cumulative pack-years among all participants [41].

### Potential shared pathways for neurotoxicity of HIV and smoking

#### The roles of neuroinflammation and brain volume changes in HIV and tobacco smoke effects on the brain

Several human and in vivo preclinical studies in transgenic mice demonstrate the potential for combined deleterious inflammatory and hormonal effects of tobacco smoking and HIV infection. In the general population, smoking showed an association with elevated levels of neuroinflammatory proteins in cerebrospinal fluid (CSF) level, including TNF-alpha, and with increased beta-amyloid and lower levels of total superoxide dismutase and nitric oxide synthase [43]. This suggests that smoking may impact the brain through excessive oxidative stress and neuroinflammation [43]. Using transgenic (Tg) rats to mimic human HIV infection, Royal et al.. studied the interaction of HIV and smoking on cognition and neuroinflammation. TNF-alpha, IL-1, and IL-6 gene expression in the frontal cortex were all increased by smoke exposure in Tg rats exposed to cigarette smoke, but not in wild type (WT) rats suggesting that HIV may increase susceptibility to proinflammatory effects of cigarette smoke [35].

Using diffusion tensor imaging and a 2 × 2 study design, Liang et al.. found that HIV and tobacco smoking had additive and synergistic adverse effects on brain diffusivities, suggesting greater neuroinflammation in PWH who smoke compared to PWH who do not [25]. The same group also demonstrated that PWH who smoke had the smallest brain volumes in several regions including the thalamus, putamen, pallidum, hippocampus, as well as total subcortical gray matter and cerebral white matter compared to PWH who do not smoke and people who smoke without HIV [26]. Furthermore, lower current CD4 counts correlated to a smaller hippocampus in PWH who smoke [26]. These volumes are important to consider as smaller regional brain volumes predict poorer cognition and greater pack-years smoked predicted smaller brain volumes particularly in PWH who smoke [26].

#### Microglial dysregulation and blood brain barrier breakdown

One of the main biological factors that has been proposed to contribute to brain effects of HIV is abnormal activation of brain microglial cells. Microglia are part of the innate immune system as the resident macrophages of the CNS, and while they are critical to maintain homeostasis in the adult brain, they also are implicated in neurodegeneration and aging [44]. Microglia are susceptible to HIV infection, and their resistance to cytotoxic effects from HIV, can allow HIV to persist in these cells [45]. HIV infection can induce the release of reactive oxygen species (ROS) that subsequently damage nerve cells to further stimulate microglial activation [46], and this can lead to accelerated brain aging [47]. Microglial ROS production is also important to consider as it can damage the blood brain barrier (BBB), and these effects can be worsened by HIV infection [45].

Microglia can become similarly dysregulated due to exposure to carcinogenic compounds found in cigarette smoke. One such compound, 4-Methylnitrosamino-1-(3-pyridyl)-1-butanone (NNK), is a tobacco specific carcinogen and leads to increased neuroinflammation and neuronal damage in mice via microglial activation [48]. NNK treatment also lead to intracellular ROS and nitric oxide release, and lower doses of NNK can activate microglia over a longer time span [48]. These effects from cigarette smoking may compound existing microglial activation from HIV infection.

Systemic inflammation from cigarette smoking related comorbidities such as chronic obstructive pulmonary disease (COPD) can also lead to microglial activation and complications in the CNS. Microglial activation in the hippocampus and suppression of synaptophysin from cigarette smoking leads to spatial working memory deficits in a mouse model of human COPD [49]. A similar study modeling COPD in mice implicates smoking-induced hippocampal microglia activation in neuroinflammation and BBB breakdown [50]. Further studies are needed to understand the cellular interactions between HIV and cigarette smoke in microglial cells.

#### The shared role of the alpha7-nicotinic receptor in smoking and HIV effects on the brain

The alpha7-nicotinic acetylcholine receptor (alpha7-nAChR) is critical to understanding the combined effects of smoking and HIV infection on the brain. The alpha7-nAChR is widely distributed in the CNS, and it serves as an anion channel whose ligands include endogenous acetylcholine and choline, as well as nicotine and HIV-gp120 [51]. This receptor allows the flow of Na^+^, K^+^ and Ca^2+^ into cells and triggers the cholinergic anti-inflammatory pathway (CAP), reviewed extensively elsewhere [51, 52]. As the name implies, this is an anti-inflammatory pathway: stimulation of the alpha7- nAChR expressed in macrophages suppresses pro-inflammatory cytokine production. In this way, acute nicotine administration may have some neuroprotective effects via engagement of the alpha7-nAChR in microglia and CD4 + T-cells [33,53,54].

These potential neuroprotective effects of nicotine nAChR binding come with caveats, however. Chronic nicotine treatment in mice increases tolerance to several actions of nicotine, leading to an increased density of brain nAChR as well as with downregulation of nicotinic receptor function [36]. This suggests that neuroprotective effects from nAChR binding in the brain may fade with continued nicotine use. Moreover, since HIV gp120 can bind irreversibly to nAChRs [55], HIV gp120 may compete with nicotine for binding to the nicotinic receptor [56]. In this way, HIV gp120 may limit the anti-inflammatory, neuroprotective effects of acute nicotine administration.

HIV may also induce nAChR mediated neurotoxicity through indirect mechanisms. Preclinical studies demonstrate that HIV binding to CD4 and coreceptors induces expression of alpha7-nAChRs in striatal neurons, leading to neurotoxicity [51, 57]. Exogenous administration of HIV gp120 induced upregulation of nAChRs, causing neuronal apoptosis in the striatum due to high intracellular calcium levels [57]. This cellular response may contribute to neuronal loss and, ultimately, neurocognitive impairment in PWH [58]. Moreover, the effects of HIV gp120 on the Cholinergic anti-inflammatory pathway (CAP) appear to extend beyond the striatum, where apoptosis occurs. HIV gp120 allows for persistent inflammation in monocyte derived macrophages through upregulation of the alpha7-nAChR and disruption of the CAP [59, 60]. While HIV gp120 does not appear to increase alpha7-nAChR levels in microglia [58], disruption of the CAP in this peripheral macrophage model warrants further investigation, since activation of blood monocytes associates with neuroinflammation in PWH [58, 61–63].

Nicotine also appears to play a role in promoting HIV persistence in the CNS. Nicotine enhances HIV transcription in microglia through the alpha7-nAChR, possibly aiding viral persistence in the CNS that could feed back into alpha7-nAChR neurotoxicity [64]. Clinically, investigators may see higher levels of viral persistence in the CNS associate with higher rates of abnormal CNS immune activation and of cognitive impairment in PWH [65]. In the periphery, HIV DNA and cell associated RNA were correlated with duration and intensity of cigarette smoking, again suggesting a relationship between smoking and loss of viral control [66].

Alpha7-nAChRs are also implicated in BBB injury, which is a shared feature of nicotine smoking and of HIV infection [67, 68]. In a mouse model of a common HIV-associated opportunistic infection, the fungal pathogen *Cryptococcus neoformans*, alpha7-nAChR knockout mice experienced significantly reduced BBB injury relative to wild type, and alpha7-nAChR and NF-kB signaling appear to mediate leukocyte transmigration across the BBB induced by the HIV-1 virotoxin gp41 and methamphetamine in vitro [67]. These results suggest that alpha7-nAChRs may play an important role in HIV associated damage to the BBB and eventual neurotoxicity. Similarly, nicotine administration appears to enhance the damaging effects of HIV gp120 on the BBB in mice and in vitro [68].

HIV infection may also cause brain injury by altering expression of the genes that encode for alpha7-nAChR genes. In a post-mortem study of PWH, gene expression of the alpha7 subunit gene, CHRNA7, and an inhibitory hybrid gene *CHRFAM7A*, were dysregulated in the basal ganglia of PWH with varying levels of NCI. Moreover, only PWH with low levels of *CHRFAM7A* transcript displayed mild neurocognitive impairment before death [69]. In a neuronal cell line, HIV gp120 downregulates *CHRFAM7A* in a dose dependent manner, and a pathophysiological dose of HIV gp120 increases the *CHRNA7*:*CHRFAM7A* ratio a time-dependent manner, with the greatest effect at 24 h after exposure [69].

Genetic polymorphisms in *CHRFAM7A* and *CHRNA7* also appear to impact the success of smoking cessation efforts. Cameli et al.. examined the relationship between genetic variation in *CHRFAM7A* and *CHRNA7* and smoking dependence and maintenance of abstinence after cessation. In a sample of 408 people seeking treatment for smoking, one of the two variants in the *CHRNA7* promoter, which is associated with decreased alpha7-nAChR activity, was associated with a greater degree of nicotine dependence [70]. All participants were then provided cognitive-behavioral counseling, and 142 of these received varenicline treatment for 12 weeks. Investigators also recruited a population control sample consisting of never and light smokers. Interestingly, an association between *CHRFAM7A* copy number and maintenance of smoking cessation was observed in those treated with varenicline, but no association was seen in the untreated participants [70]. Given the context dependent effects of gene expression on smoking cessation and nicotine dependence, future studies should focus on genetic variation in the in *CHRFAM7A* and *CHRNA7* genes in PWH seeking treatment for smoking.

Taken together, these data highlight the importance of further investigating the shared role alpha7-nAChR in HIV associated NCI and the neuropathology arising from tobacco smoking. Understanding this shared role not only advances our understanding of how comorbidities can influence neurocognitive function in PWH, but also to explore this receptor as a therapeutic target. While the evidence suggests exciting potential for this receptor as a target for treatment, much of this evidence is preclinical, and longitudinal, translational and clinical studies are needed to confirm the preclinical findings.

## Conclusions and future directions for research

HIV infection coupled with tobacco smoking appears to lead to combined adverse effects on the brains of PWH. PWH who smoke may experience worsened combined effects of HIV infection and smoking on cognitive function and decline, and this may be exacerbated by increased exposure to cigarette smoking [25–29, 31, 39]. While some studies find nonsignificant additional impairment in PWH who smoke relative to PWH who do not smoke these studies tend to be limited in design as previously discussed (Table 1) [40–42].

PWH are also less likely to quit smoking compared to the general population, though PWH may be more likely to initiate cessation with adequate access to cessation resources [3–5]. While biological mechanisms are one aspect to consider for smoking cessation treatment in PWH, we must also emphasize the importance of psychosocial factors. For example, smoking in PWH is also associated with lower levels of ART adherence and may serve as a form of self-medication for anxiety and depression in PWH [2, 10]. PWH who smoke tend to have higher levels of anxiety and depression than PWH who do not smoke and those who smoke without HIV [31]. The importance of treating anxiety and depression for smoking cessation in PWH is further supported when considering how nicotine replacement therapy integrated with cognitive behavioral therapy can improve cessation outcomes in PWH [71].

Additionally, the adverse effects of diminished ART adherence may be compounded through smoking’s metabolic effects on ART metabolism. Smoking can potentially limit the effectiveness of ART through metabolic pathways, particularly through cytochrome P450 enzymes (CYPs). For example, a population pharmacokinetics study of treatment-naive PWH implicates smoking as a predictor of elevated apparent clearance of dolutegravir (DTG) [72]. To elucidate the mechanism of this altered metabolism, Zhu et al.. examined DTG metabolite formation and found the extrahepatic CYPs, CYP1A1 and CYP1B1, to be important in DTG metabolism; both of these enzymes are induced by cigarette smoking [73].

Considering alternative forms of nicotine use, there is limited evidence to support vaping, or electronic cigarettes, as an effective smoking cessation method or harm reduction strategy for PWH who smoke [74]. One barrier for vaporized nicotine (VN) as an alternative is the existing high rate of simultaneous combustible cigarette use among PWH at 51% [75]. Also, despite possibly being used as a form of self-medication, psychiatric symptoms including panic disorder and depression persist among PWH who use VN [75]. Prospects for VN as a smoking cessation method for PWH seem even less likely when considering the opinions of PWH who smoke [76]. However, 11% of PWH reported ever using VN, so more clinical and pre-clinical investigation is warranted to understand how VN may interact with HIV to affect brain and CNS health [75].

Future studies of potential shared microglial pathophysiology in smoking and HIV should expand beyond only considering the interaction between nicotine and HIV on microglia to cigarette smoke and HIV [53]. These interactions may be region specific, and the hippocampus may be of importance based on previous studies examining the effects of smoking on microglia [48, 50]. Associations between smoking in PWH and CSF biomarkers of neuronal injury such as neurofilament light chain (NF-L), and of immune activation, such as neopterin, combined with data on nAChR function and polymorphisms could also prove fruitful avenues of investigation.

Finally, there are important considerations when constructing cohorts as research on smoking in PWH emphasizes patient-facing research. PWH are a diverse group, including people with marginalized identities, including racial and ethnic minorities, living in an LMIC, lower socioeconomic status, being a part of the LGBTQ + community, and other groups that are sometimes excluded from research studies. Studying smoking cessation in minority and marginalized groups of people with HIV is crucial because these populations often face unique social, economic, and health disparities that can exacerbate the challenges of quitting smoking [77]. These groups may have higher rates of smoking and are more likely to encounter barriers to accessing effective cessation resources, such as tailored interventions and support services [78]. By focusing on these populations, research can address critical gaps in knowledge, develop culturally and contextually relevant interventions, and ultimately reduce health disparities, improving overall outcomes for individuals and communities disproportionately affected by both smoking and HIV.

**Table 1:** A summary of the core clinical studies evaluating the outcome of living with HIV and smoking status on neurocognitive assessment. Each study includes a description of the cohort, the main findings, and limitations that may explain why results diverge. Studies focus on people with HIV (PWH), people without HIV (PWoH), women with HIV (WWH), and/or women without HIV (WWoH)

Clinical Study Characteristics				
Author	Year	Design	Main Findings	Limitations
Bryant et al.	2013	Only PWH grouped by smoking status n_current_ = 74 n_former_ = 15 n_never_ = 26	Smoking associated with neurocognitive impairment in memory, learning, and global cognitive functioning	Groups varied by HCV infection, alcohol use, and education. Multivariate analysis shows findings non-significant. Authors suggest low statistical power.
Monnig et al.	2016	Men who have sex with men (MSM) living with HIV and history of heavy drinking. *n* = 124	Smoking significantly negatively associated with verbal learning and processing speed. Smoking had significant effects on cognition in all regression models	Possibly limited generalizability because only focused on MSM with HIV and history of heavy drinking. Observational and cross sectional
Chang L. et al.	2017	PWH and PWoH who do and do not smoke. n_PWH who smoke_ = 22 n_PWH who do not smoke_ = 29 n_PWoH who smoke_ = 26 n_PWoH who do not smoke_ = 29	PWH scored lower in all cognitive domains tested and the lowest scores were among PWH who smoke. PWH who smoke performed the worst on IGT and WCST which assess impulsiveness and decision making.	Cross sectional design and small sample size.
Harrison et al.	2017	Comparing people who smoke with and without HIV n_PWH who smoke_ = 103 n_PWoH who smoke_ = 70	PWH who smoke performed worse than those without HIV on working memory, processing speed, and had higher intra-individual variability. HIV status improved ROC model	Authors did not confirm HIV- serostatus, no PWoH who do not smoke, recruitment from two different clinical trials with different selection criteria.
Wojna et al.	2007	Women with and without HIV (WWH, WWoH) grouped by smoking status n_WWH current smoking_ = 15 n_WWoH current smoking_ = 4 n_WWH History of Smoking_ = 24 n_WWoH History of Smoking_ = 7	Higher plasma VL in WWH who smoke but not seen in CSF. No association between cognitive impairment and current or past history of smoking. When analyses restricted to women with HIV, history of smoking associated with better executive cognitive domain performance	Cross Sectional and a brief neuropsychological testing battery with only X cognitive domains. Also, a very small sample size. WWoH who smoke *n* = 4. Women with HIV significantly older. May lack statistical power to uncover effect of smoking on cognition in women with HIV
Akhtar-Khaleel et al.	2017	MACS cohort of MSM over 50 with and without HIV n_PWH currently smoke_ = 65 n_PWoH currently smoke_ = 120 n_PWH formerly smoked_ = 100 n_PWoH formerly smoked_ = 172 n_PWH never smoked_ = 53 n_PWoH never smoked_ = 72	No difference in rate neurological decline between smoking groups in both PWoH and PWH. Small effect of cumulative smoking on neurological decline in PWoH only.	Brief Neuropsychological testing battery only analyzing cognitive flexibility and speed of information processing. These participants were also survivors of the original pandemic which may not be representative of most PWH today.
Tsima et al.	2018	Retrospective cohort study at UPenn CFAR n_PWH who smoke_ = 1486 n_PWH who do not smoke_ = 1547	No difference in clinically significant neurocognitive impairment. PWH who smoke had 12% higher risk of clinically significant neurocognitive impairment than those who do not smoke but not significant.	Very brief neuropsychological testing battery consisting of three tasks: (1) Counting sequentially from 1–20, (2) Reciting the alphabet, (3) Alternating numbers and letters sequentially for 30 s. Limited information on smoking history. Groups not well matched for baseline exposures

## Data Availability

No datasets were generated or analysed during the current study.
